# Astroglial conditional *Slc13a3* knockout is therapeutic in murine Canavan leukodystrophy

**DOI:** 10.1002/acn3.52010

**Published:** 2024-01-28

**Authors:** Vanessa L. Hull, Yan Wang, Jennifer McDonough, Meina Zhu, Travis Burns, Najmah Al Ramel, Ali Dehghani, Fuzheng Guo, David Pleasure

**Affiliations:** ^1^ Department of Neurology UC Davis School of Medicine Sacramento California USA; ^2^ Shriners Hospital for Children Sacramento California USA; ^3^ Department of Physiology David Geffen School of Medicine, UCLA Los Angeles California USA; ^4^ Department of Biological Sciences Kent State University Kent Ohio USA

## Abstract

Canavan disease is a leukodystrophy caused by *ASPA* mutations that diminish oligodendroglial aspartoacylase activity, and is characterized by markedly elevated brain concentrations of the aspartoacylase substrate N‐acetyl‐l‐aspartate (NAA) and by astroglial and intramyelinic vacuolation. Astroglia express NaDC3 (encoded by *SLC13A3*), a sodium‐coupled transporter for NAA and other dicarboxylates. Astroglial conditional *Slc13a3* deletion in aspartoacylase‐deficient Canavan disease model mice (“CD mice”) reversed brain NAA elevation and improved motor function. These results demonstrate that astroglial NaDC3 contributes to brain NAA elevation in CD mice, and suggest that suppressing astroglial NaDC3 activity would ameliorate human Canavan disease.

## Introduction

Canavan disease is a recessively inherited early onset leukodystrophy caused by inactivating *ASPA* mutations that diminish oligodendroglial aspartoacylase activity.^1^ The concentration of the aspartoacylase substrate N‐acetyl‐l‐aspartate in brain ([NAA_B_]) is elevated in these patients, who fail to attain or maintain motor and intellectual milestones, are often neonatally megalencephalic, and frequently develop seizures.^1^, ^2^ Neuroimaging reveals brain white matter signal abnormalities consistent with cytotoxic edema, and autopsy demonstrates astroglial and intramyelinic vacuolation.^3^, ^4^ Mice homozygous for the *Aspa* nonsense mutation *nur7* (“CD mice”) lack detectable aspartoacylase, demonstrate a typically twofold or greater elevation in [NAA_B_], and develop motor deficits by postnatal day 21.^5^

NaDC3, encoded by *SLC13A3* (*Slc13a3* in mice), is a plasma membrane Na^+^‐coupled dicarboxylate transporter that can mediate intracellular accumulation of NAA and Na^+^ from the medium by cultured rat astroglia.^6^, ^7^ *Slc13a3* is also expressed by meningeal cells and renal proximal tubular epithelium.^6^, ^8^ To explore the specific pathophysiological significance of astroglial NaDC3 in Canavan disease, we examined the effects of astroglial *Slc13a3* conditional knockout on [NAA_B_] and motor function in young adult CD mice.

## Methods

**Figure 1:**
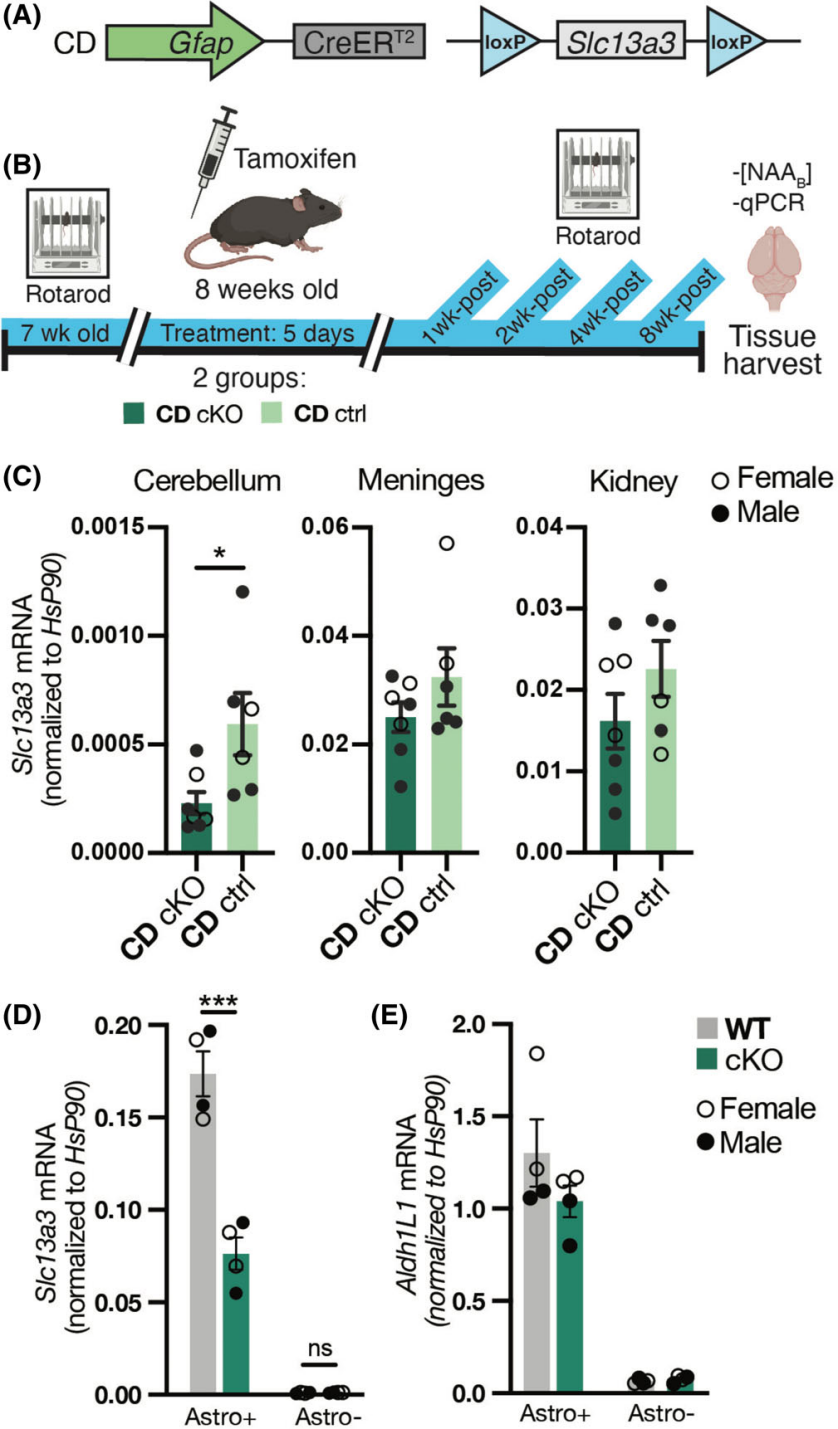
Panel A: *Slc13a3* conditional deletion construct. Panel B: Experimental design. Panel C: qRT/PCR *Slc13a3* mRNA results in cerebellum, meninges, and kidney of CDcKO (dark green) and CDctrl (light green) mice, normalized to Hsp90 mRNA. Panel D: Astrocytes were isolated using MACS with Anti‐ACSA‐2 MicroBeads (ACSA‐2: astrocyte cell surface antigen‐2) according to the manufacturer's instruction (Miltenyi Biotec) The flow‐through, devoid of ACSA‐2‐positive astrocytes, was collected as the astrocyte‐depleted fraction. Panel E: Quantitative PCR was used to quantify the astrocyte marker *Aldh1L1* mRNA^9^ (forward primer 5′‐CAGGAGGTTTACTGCCAGCTA‐3′ and reverse primer 5′‐CACGTTGAGTTCTGCACCCA‐3′) and *Slc13a3* mRNA (see Methods) in the astrocyte‐enriched and astrocyte‐depleted fractions. Each circle represents results in an individual mouse (solid circles male and open circles female). Column heights represent means, and vertical brackets denote SEMs. *p = 0.0265, ***p = 0.0003, Student's *t*‐test.

*Slc13a3* ^*flox*^ mice were derived from *Slc13a3* knockout first mice (MMRRC.049665‐UCD) by crossing with Flp1 deleter mice (Jackson Laboratory #009086). The *Slc13a3* ^*flox*^ mice were then crossed with *Aspa* ^*nur7*^ mice (Jackson Laboratory #008607) and with *GFAPcreER* ^*T2*^ mice (Jackson Laboratory #012849) to yield the “CDcKO” mice used in this study, which were homozygous for *Aspa* ^*nur7*^ and *Slc13a3* ^*flox*^ and carried one copy of the *GFAPcreER* ^*T2*^ transgene (Fig. 1A). Control (“CDctrl”) mice were bred to also be homozygous for *Aspa* ^*nur7*^, but to lack either *GFAPcreER* ^*T2*^ or *Slc13a3* ^*flox*^ alleles. Mouse genotypes were validated by qPCR. All mice were maintained on a C57BL6 background in an AAALAC‐certified vivarium. Both male and female mice were employed. All animal procedures were conducted in accord with a UC Davis IACUC‐approved protocol.

An accelerating rotarod apparatus was used to assess motor function in non‐tamoxifen‐treated CD mice, tamoxifen‐treated CD/*GFAPcreER* ^*T2*^ */Slc13a3* ^flox/flox^ mice (“CDcKO” mice), and tamoxifen‐treated CDctrl mice just before and at intervals after a 5 day course of tamoxifen (1.5 mg/50 μL sunflower oil/day) (Fig. 1B).^10^ Eight weeks post‐tamoxifen, the mice were euthanized; meninges were carefully dissected away from underlying brain; and cerebella, forebrain, meninges, and kidneys were separately flash‐frozen and stored at −80°C. *Slc13a3* recombination in cerebellar parenchyma, meninges, and kidney was assessed by qPCR, using the *Slc13a3* cDNA primers (forward primer 5′‐TCTCAGTGTAAGAAGCGC‐3′ and reverse primer 5′‐CACTGCCTGGGTTCAAAGTC‐3′). Results were normalized to *Hsp90 mRNA* (forward primer 5′‐AAACAAGGAGATTTTCCTCCGC‐3′ and reverse primer 5′‐CCGTCAGGCTCTCATATCGAAT‐3′). Forebrain samples from euthanized 16 week old CDcKO and CDctrl mice were assayed for NAA by HPLC^8^ or enzymatically dissociated prior to magnetic activated cell sorting (MACS)^11^ to prepare astrocyte‐enriched and astrocyte‐depleted cell fractions for qPCR quantifications of *Slc13a3* mRNA and of *Aldh1L1* mRNA. Cryostat forebrain and cerebellar sections from 16‐week‐old wild‐type, CDcKO and CDctrl mice were employed to quantify cerebellar vacuolation.

## Results

Tamoxifen activation of *GFAPcreER* ^*T2*^ elicited a 61% reduction in *Slc13a3* mRNA abundance in cerebellar parenchyma of CDcKO mice, but did not significantly alter cerebellar parenchymal *Slc13a3* mRNA abundance in CDctrl mice, nor in the meninges and kidneys of either CDcKO or CDctrl mice (Fig. 1C). Magnetic activated cell sorting was used to prepare astroglia‐enriched (“astro^+^”) and astrocyte‐depleted (“astro^−^”) cell fractions from forebrains of 16‐week‐old wild‐type (WT) mice. Abundances of both *Slc13a3* mRNA and mRNA encoding the astrocyte marker Aldh1L1^9^ were markedly greater in the astro^+^ fraction than in the astro^−^ fraction, and *Slc13a3* mRNA abundance was 56% lower in the astroglial fraction from tamoxifen‐treated than from non‐tamoxifen‐treated *GFAPcre* ^ERT2^/*Slc13a3* ^flox/flox^ mice (Fig. 1D).

**Figure 2:**
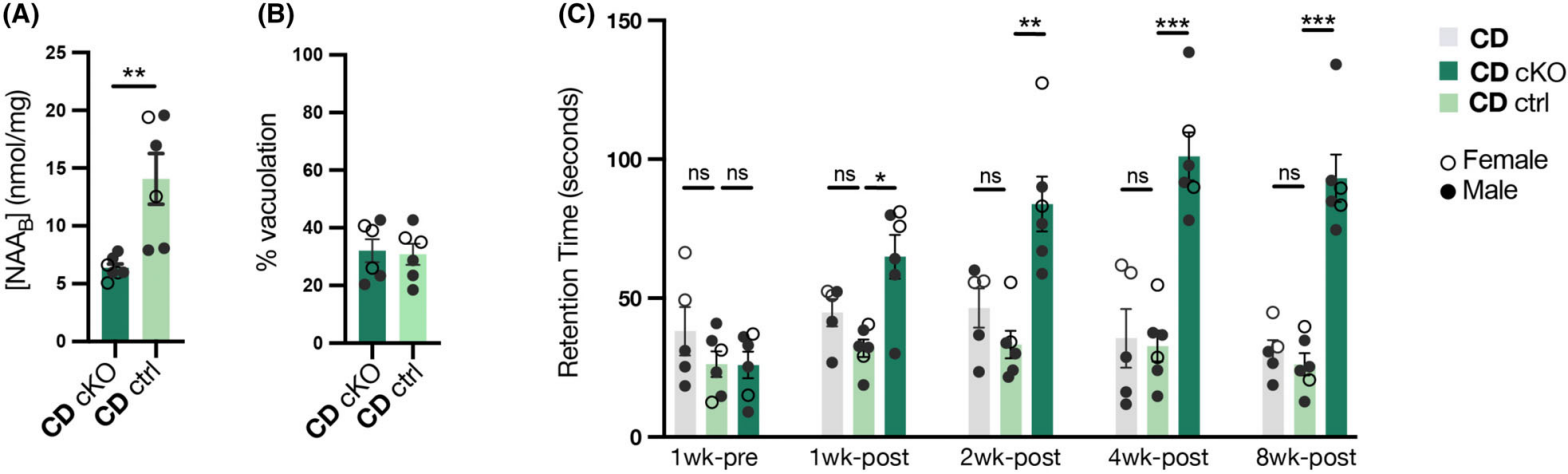
Panel A: [NAA_B_] of CDcKO (dark green) and CDctrl (light green) mice. **p = 0.0031, Student's *t*‐test. Panel B: Cerebellar vacuolation in CDcKO and CDctrl mice, showing no significant difference between the two groups. Panel C: Accelerating rotarod retention times of CDcKO and CDctrl mice. Column heights represent means, and vertical brackets denote SEMs. *p < 0.05; **p = 0.0043; ***p < 0.0001, comparisons by two‐way repeated measures ANOVA.

At age 16 weeks, [NAA_B_] in the tamoxifen‐treated CDcKO mice was 55% below that in the tamoxifen‐treated CDctrl mice (Fig. 2A), but the extent of cerebellar vacuolation in the CDcKO mice did not differ significantly from that in the CDctrl mice (Fig. 2B). Accelerating rotarod retention times in CDcKO mice had significantly increased by 1 week after completion of tamoxifen administration, and remained substantially elevated through 7 additional weeks of observation, whereas accelerating rotarod retention times in CD mice not treated with tamoxifen and in CDctrl mice treated with tamoxifen remained consistent throughout the entire period of testing (Fig. 2C).

## Discussion

Prior studies have demonstrated that lowering [NAA_B_] by inhibiting neuronal NAA synthesis can prevent or reverse brain vacuolation and neuronal dendritic shortening in CD mice.^12^, ^13^, ^14^ More recently, whole body NaDC3 ablation achieved by constitutive *Slc13a3* deletion was also found to prevent [NAA_B_] elevation and leukodystrophy in CD mice.^8^ But that study did not illuminate the specific role of astroglial NaDC3, versus NaDC3 encoded by meningeal and renal *Slc13a3*,^8^ in mediating [NAA_B_] elevation and eliciting clinical neurological deficits in this murine Canavan disease model. We addressed this issue by testing the effects of astroglial conditional *Slc13a3* deletion in young adult CD mice on [NAA_B_], brain vacuolation, and motor function.

Gene recombination elicited by *GFAPcreER* ^*T2*^ in the adult murine CNS is restricted largely to astroglia.^15^ In the present study, the abundance of *Slc13a3* mRNA was substantially lower in whole cerebellar parenchyma of 16‐week‐old CDcKO than CDctrl mice, but was not lowered in CDcKO meninges or kidneys. MACS results indicated that *Slc13a3* mRNA expression in brain parenchyma was confined largely to astroglia, and was diminished in astroglia by more than 50% by CDcKO. We concluded, therefore, that mouse brain *Slc13a3* knockdown elicited in tamoxifen‐treated CD/*GFAPcreER* ^*T2*^ */Slc13a3* ^flox/flox^ mice was largely or wholly confined to astroglia.

The substantially lower [NAA_B_] in the 16 week old CDcKO than CDctrl mice supports the concept that astroglial NaDC3 activity contributes to brain NAA elevation in Canavan disease. Though astroglial conditional *Slc13a3* knockout initiated at age 8 weeks did not yield a reduction in the extent of cerebellar vacuolation at age 16 weeks, accelerating rotarod analysis demonstrated a rapid and long‐lasting improvement in motor function in the CDcKO mice. A similarly rapid initial improvement in motor function was previously observed in young adult CD mice when [NAA_B_] was lowered by inhibiting brain NAA synthesis via intrathecal administration of an *Nat8l* antisense oligonucleotide.^14^ Together, these observations strengthen support for the pathophysiological significance of elevated [NAA_B_] in Canavan disease.

Are *SLC13A3* and NaDC3 suitable targets for treating Canavan disease? Constitutive *Slc13a3* deletion in wild‐type mice has no obvious phenotypic effects other than increasing urinary NAA output,^8^ but, in humans, mutations that inactivate both alleles of *SLC13A3* increase susceptibility to viral infection‐triggered acute reversible leukoencephalopathy.^16^ Thus, measures designed to suppress global, or astrocyte‐specific, *SLC13A3* expression and NaDC3 activity in infants and children with Canavan disease may prove useful in limiting [NAA_B_] elevation and preventing or ameliorating their neurological deficits, but might also increase their risk for developing reversible leukoencephalopathy.

## Author Contributions

VLH and DP wrote the first draft. All authors provided critical review and feedback and approved the final draft. VLH, YW, JM, and FG provided statistical analysis, VLH, YW, and DP designed the work. VLH, YW, JM, MZ, TB, NR, AD and DP performed and interpreted the studies.

## Conflict of Interest

The authors declare no conflict of interest.
